# Sports and Weight Control in Children

**DOI:** 10.7759/cureus.53731

**Published:** 2024-02-06

**Authors:** Artemii Lazarev, Sahil Nath, Christine Q Nguyen, Anna M Demian, Raphael A. O Bertasi, Tais G. O Bertasi, George G. A Pujalte

**Affiliations:** 1 Internal Medicine, Mount Sinai Hospital Chicago, Chicago, USA; 2 Family Medicine, Mayo Clinic, Jacksonville, USA; 3 Internal Medicine, Mount Sinai Morningside West, New York, USA

**Keywords:** physical activity, weight control, adolescent, sports, pediatric obesity

## Abstract

Pediatric obesity is a global concern with distressing comorbid conditions, including mood disturbance, cardiovascular changes, endocrine imbalance, liver disease, sleep apnea, and orthopedic conditions. The primary treatment of this condition includes physical activity. Participating in organized sports has been shown to reduce weight and the complications of pediatric obesity more effectively than individual exercise.

## Introduction and background

Over the last 20 years, the rate of obesity has rapidly increased on a global scale, with the US at the forefront [1]. In the US, pediatric obesity affects 17.4% of the population aged 2 to 17 [2]. In the pediatric population, the Centers for Disease Control and Prevention (CDC) define overweight as body mass index (BMI) between the 85th and 95th percentile and obesity as greater than or equal to the 95th percentile on their clinical weight-for-age growth charts. Risk factors for the development of obesity can be genetic, environmental, sociocultural, economic, familial, and psychological [3]. Ideally, obesity is prevented through an active lifestyle and healthy eating. However, management and adherence to treatment once obesity occurs can be difficult. The Physical Activity Guidelines for Americans recommend that children participate in at least 60 minutes of at least moderate-intensity exercise daily, but unfortunately, only about 24% of children aged 6 to 17 are meeting this goal [3]. The management and treatment of pediatric obesity is vital, as its comorbid conditions, including hypertension, dyslipidemia, insulin resistance, and nonalcoholic fatty liver disease (NAFLD), can continue into adulthood and lead to increased morbidity and mortality [4]. While medical and surgical therapies are implemented in some situations, the mainstay of treatment remains lifestyle modification with diet and physical activity.

## Review

Youth sports and society

Youth sports have been a part of our society since the beginning of the twentieth century. The goal of youth sports is to build character, occupy time, and help with the transition into adulthood. Since the early days, youth sports have been sponsored by various organizations, including schools and social clubs such as the Young Men’s Christian Association, Young Women’s Christian Association, Boys & Girls Clubs of America, Boy Scouts, and Girl Scouts. A great example of organized youth sports is Little League Baseball. It was created in 1939 by Carl Stotz currently uniting two million children in 50 US states and more than 80 countries [5].

About 50% of children participate in some type of sport worldwide, and the youth sports industry is valued at over $15 billion [6]. Children participating in sports have been shown to have increased physical strength and abilities, as well as improved mental health and decreased risk of depression. Participation in youth sports has also led to decreased substance abuse, better behavior, safer sexual practices, and better overall health due to increased activity and improved diet. All these benefits carry into adulthood, as many of these sports and activities can lead to lifelong enjoyment. The potential social benefits of youth sports participation are team building, friendship, and increased popularity in social groups [6].

However, there are also potential downsides to participation in youth sports. There are instances where children have dropped out of youth sports due to negative experiences, including excessive training demands, long practice hours, unreasonable expectations, competition anxiety, and fear of injury [6]. Positive support from coaches and parents is crucial in helping children overcome such barriers and can lead to success and favor the more beneficial outcomes of youth sports.

Problems with pediatric obesity

Pediatric obesity is associated with many psychological, physiological, and social consequences [3]. These consequences are strongly related to each other and contribute to the development of obesity, meaning the connection between pediatric obesity and its consequences is often bidirectional [7]. Psychological consequences include depression, anxiety, self-esteem issues, body dissatisfaction, and eating disorders. Physiological and pathophysiological consequences include high blood pressure, lipid dysregulation, insulin resistance, and high mechanical load on joints [8] while social consequences include marginalization and discrimination [9].

In a meta-analysis by Sutaria et al., the authors demonstrated that children with obesity have higher odds of depression compared with children with a healthy weight (odds ratio, 1.32; 95% CI, 1.17-1.50), with odds of depression being higher for girls with obesity (odds ratio, 1.44; 95% CI, 1.20-1.72) [10]. The authors also showed that this risk persists into adulthood [10]. Another meta-analysis showed that children with obesity and adolescents are more likely to suffer from depression and female and non-Western children are at higher risk of depression development [11]. Lindberg et al. reported that though both girls and boys have higher risk of anxiety and depression, girls were more affected (adjusted hazard ratio, 1.43 (95% CI, 1.31-1.57; P<.0001) vs adjusted hazard ratio, 1.33 (95% CI, 1.20-1.48; P<.0001), respectively) [12]. However, this connection might be bidirectional, as a previous study in adults showed that depression might be predictive of developing obesity [13], and another study suggested there might be a similar association in children and adolescents [3].

Pediatric obesity was also linked to body dissatisfaction and low self-esteem. Interestingly, the association between BMI and body satisfaction differs between boys and girls. In girls, increasing BMI linearly amplifies body dissatisfaction, while in boys, the curve is U-shaped [14]. The fact that low BMI is also associated with body dissatisfaction is explained by the cultural importance of both muscularity and leanness for boys and men [14]. Furthermore, up to 10% of boys with obesity and 20% of girls with obesity have low self-esteem [15]. Delgado Floody et al. showed that low self-esteem and very low self-esteem among 12-year-old children were associated with being overweight and obese [16]. Notably, Gong et al. demonstrated that children who successfully reduced their weight might have had better self-esteem than children who had never been overweight or obese [17].

Obesity is a well-known factor contributing to the development of numerous physiologic and medical conditions in adults [8]. Studies have shown similar effects in children; pediatric obesity appears to affect every organ system and has multiple health consequences, including atherosclerosis, hypertension, dyslipidemia, insulin resistance, prediabetes, type 2 diabetes, and NAFLD [8,18,19].

Among the most concerning consequences of obesity are cardiovascular risks [20]. Atherosclerosis and hypertension are strongly associated with pediatric obesity [20], specifically the development and progression of fatty streaks, seen on autopsy [21], as well as various surrogate markers of atherosclerosis [22]. The pathophysiology of atherosclerosis and subsequent hypertension include increased preload, ectopic fat accumulation in the myocardium, and vascular damage due to inflammation [20,23]. This may lead to left ventricular hypertrophy early in life [24]. Insulin and leptin further contribute to atherosclerotic plaque formation increasing arterial stiffness [23].

Pediatric obesity contributes to the development of insulin resistance with subsequent prediabetes and type 2 diabetes. In fact, high insulin level is the most common biochemical change observed in obesity [8]. Insulin resistance with hyperinsulinemia followed by insulin secretion abnormalities and fasting hyperglycemia increases the risk of prediabetes and diabetes. Pediatric diabetes prevalence has consistently risen, and the degree of this rise varies among ethnic and socioeconomic groups [25]. Per 1,000 youths aged 19 years or younger, diabetes prevalence increased from 1.48 in 2001 to 2.15 in 2017, mainly in non-Hispanic Black and non-Hispanic White populations. Furthermore, type 2 diabetes prevalence per 1,000 youths aged 10 to 19 years increased from 0.34 in 2001 to 0.67 in 2017, mainly in non-Hispanic Black and Hispanic populations [25]. Diabetes is a separate cardiovascular risk factor that significantly increases the risk of cardiovascular events in the future [20]. Studies show that insulin resistance results from high levels of free fatty acids and proinflammatory factors in the blood. Both factors are associated with visceral, intraperitoneal, and subcutaneous fat deposits in the body [26,27]. With underlying genetic or epigenetic predisposition [28], insulin resistance rapidly progresses to prediabetes or type 2 diabetes. This progression occurs faster in children than in adults and is the result of a more rapid decline in β-cell function [29].

Pediatric obesity also causes changes in hormonal systems [8] because it is widely associated with polycystic ovary syndrome (PCOS) [30]. The prevalence of PCOS in adolescents ranges from 3.39% to 11.04% [31]. It is hypothesized that obesity alters the mitochondrial function in the oocyte through lipotoxicity and free fatty acid excess, producing a toxic effect in the ovary [32] and the release of bioactive molecules, called adipokines, from the adipose tissue [32]. The other possible risk of PCOS development in girls with obesity is chronic inflammatory states [33]. PCOS is associated with hyperandrogenism [34] and infertility [35] and must be considered to protect the fertility of the patients [30].

Thyroid abnormalities, particularly thyroid-stimulating hormone elevation with normal or slightly elevated free thyroxine and triiodothyronine, are also associated with pediatric obesity [36]. It remains unclear if higher levels of thyroid-stimulating hormone are the adaptation to increased metabolic rate in children with obesity or if it is a causative trigger for obesity [36].

NAFLD and its progressive form, nonalcoholic steatohepatitis, appear to be the most common gastrointestinal and liver diseases among the pediatric population [2], with an estimated prevalence of 36.1% in children and adolescents with obesity [37]. NAFLD is associated with cirrhosis and other extrahepatic morbidities. Healthy eating and physical activity are the only prevention and treatment measures for pediatric NAFLD [37].

The risk of obstructive sleep apnea is higher in children with obesity, increasing their risk for cardiovascular disease, abnormal behaviors, neurocognitive dysfunction, growth abnormalities, and inflammation [38,39]. The prevalence of obstructive sleep apnea with pediatric obesity ranges from 0% to 5.7%, with obesity being an independent factor in the development of the disease [39].

Pediatric obesity is associated with several orthopedic adverse conditions, including genu varum, genu valgum, and slipped capital femoral epiphysis [40]. Obesity affects the pattern and severity of orthopedic injury [41], and the development of bone pathology in pediatric obesity is complex. Adipose tissue secretes leptin, which inhibits cortical bone formation and increases bone resorption [42]. Chronic inflammation in pediatric obesity also induces bone resorption, leading to osteopenia and osteoporosis [43]. Vitamin D deficiency, highly prevalent in children with obesity, also contributes to decreased bone density [44]. Children with obesity tend to have more severe and complicated fractures and more extremity fractures than children with healthy weight, and they are more likely to need surgical treatment [45,46]. These trends are supported by the idea that with the increase in body mass, the force generated from a similar mechanism of injury increases as well, leading to more severe injury patterns in patients with obesity [41].

Effects of sports on lowering obesity

The effects of participation in specific sports on obesity have been well-documented [47-49]. Physical activity reduces body fat content in prepubertal children [6]. The impact of participation in various sports on obesity is influenced by their specific qualities and characteristics. This portion explores the various effects of participation in specific sports on human physiology, as well as the differing effects of participation in organized sport vs individual exercise.

Participation in sports impacts the following measurements: BMI, fat mass and fat-free mass, muscle mass, and osteogenesis. Muscles consume energy (calories), and as muscle mass increases, so too does energy expenditure, helping reduce excess body fat and weight. Additionally, muscle mass makes metabolism efficient [50]. BMI indirectly measures body fat based on height and weight; however, BMI has inherently low specificity. Accurate assessments of fat mass and fat-free mass can increase the capacity to identify the effect of adiposity excess and the effectiveness of interventions to reduce obesity levels [51]. According to Bailey et al., 26% of adult bone mineral content is achieved between the ages of 12 and 14 [52]. Therefore, using physical activity for proper bone mass accumulation may be essential to reduce the risk of fractures in adulthood [51].

The literature suggests that participation in organized sports is associated with healthier eating habits, favorable motor development, and other developmental factors, such as proper body mass, healthy BMI, and academic achievements, as compared to individual exercise [1]. Additionally, participation in organized sports is positively associated with mental health, perceived health and well-being, self-concept, self-esteem, self-regulation, self-efficacy, competence, social skills, enjoyment, satisfaction, connectedness, belonging, interdependence, and group cohesion [53]. This is noteworthy as research shows the importance of behavioral change for longevity in weight reduction [50].

In practice, pediatric organized sports should prioritize fun and skill development rather than winning. It is important to create a positive, age-supportive, and inclusive environment for children of all body types, abilities, and ages. Programs should offer a variety of sports to allow children to discover their interests. Organized sports can include both individual and team sports, with examples such as walking, dancing, swimming, biking, yoga, and football (the latter two will be discussed in detail below). Ultimately, the best sport or activity for an obese child will depend on their interests and physical abilities. Positivity should be embraced by providing support, constructive feedback, and encouragement, with the possible involvement of family members. Children should begin with fundamental movement skills such as running, jumping, and swimming and progress gradually at their own pace.

An example of an individual sport would be yoga. Participation in yoga has been shown to have a significant impact on pediatric obesity [54]. Continuous yoga, which is a cyclic yoga practice with minimal rest periods, had a statistically significant effect on decreasing BMI and body fat mass and increasing muscle mass compared to the control group [55]. A study in Finland revealed that participation in yoga was associated with reduced total cholesterol, triglycerides, blood pressure, heart rate, and BMI in participants with cardiovascular disease [56]. Thus, yoga may be used as an alternative therapy for obesity prevention and health promotion in adolescents with obesity [56]. Different yoga postures, especially forward and backward bending and twisting, are efficient in reducing fat in the body. Additionally, yoga does not require machines or a large amount of physical space.

Breathing is another unique aspect of yoga that brings a holistic approach to health. Breathing exercises involve the manipulation of breath as a dynamic bridge between the body and mind [57]. This is especially important considering the dynamic nature of obesity involving both body and mind [58]. Breathing and yoga treatment programs must include a behavioral component to permanently change the nutrition and physical exercise habits of children or adolescents who are obese [59].

An example of a team sport would be football. Participation in youth football is associated with increased lean body mass and whole-body bone mineral content and decreased fat mass. More specifically, the aerobic demands of football lead to higher fat oxidation during exercise and greater fat loss compared to less intense activities [53]. Football also includes a multitude of high-speed actions, including sprints, turns, and jumps. The impacts generated by these movements performed at an early age improve bone development by leading to increased bone mineral density during growth [51].

It is important to emphasize that organized sports should be combined with dietary interventions for their full effect. A healthy diet is extremely important in the management of obesity, and although it is beyond the scope of this article, we encourage readers to familiarize themselves with dietary approaches to the management of pediatric obesity [60,61].

## Conclusions

Pediatric obesity presents significant risks that can be mitigated through various interventions. When combined with physical activity, proper nutrition, and behavioral treatments, involvement in various sports has proven to be an effective strategy for weight loss. Participation in organized sports is one of the most promising approaches to addressing pediatric obesity.
